# Supplementary figures and images for: Molecular determinants of complexin clamping and activation function
Source: eLife. 2022 Apr 20;11:e71938. doi: 10.7554/eLife.71938 (PMC9020821; doi:10.7554/eLife.71938)

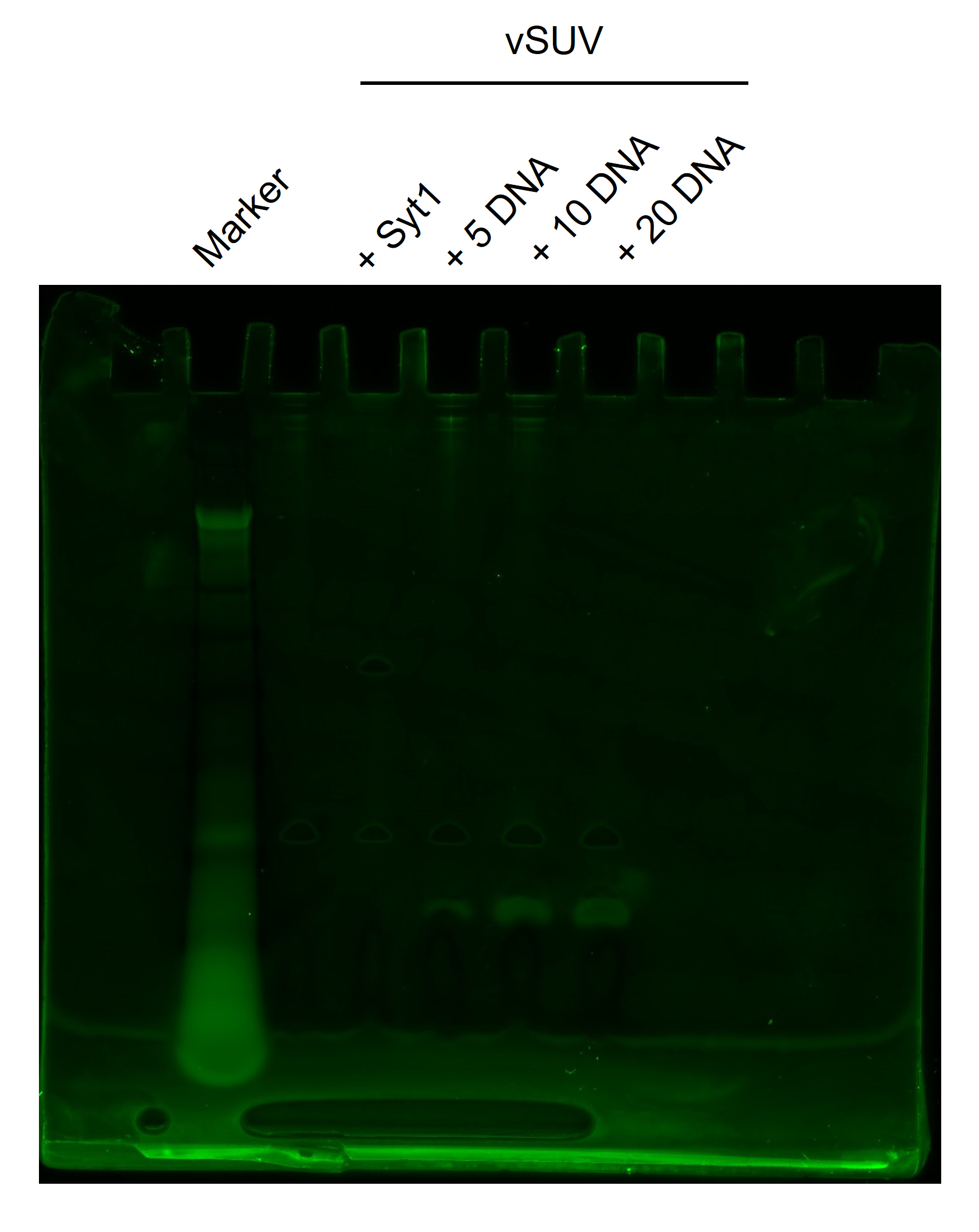

Supplement: Source data 1. [file elife-71938-data1.zip › Bera et al_Figure 1 Supplement 3_Source Gel 2_Labelled.tif]

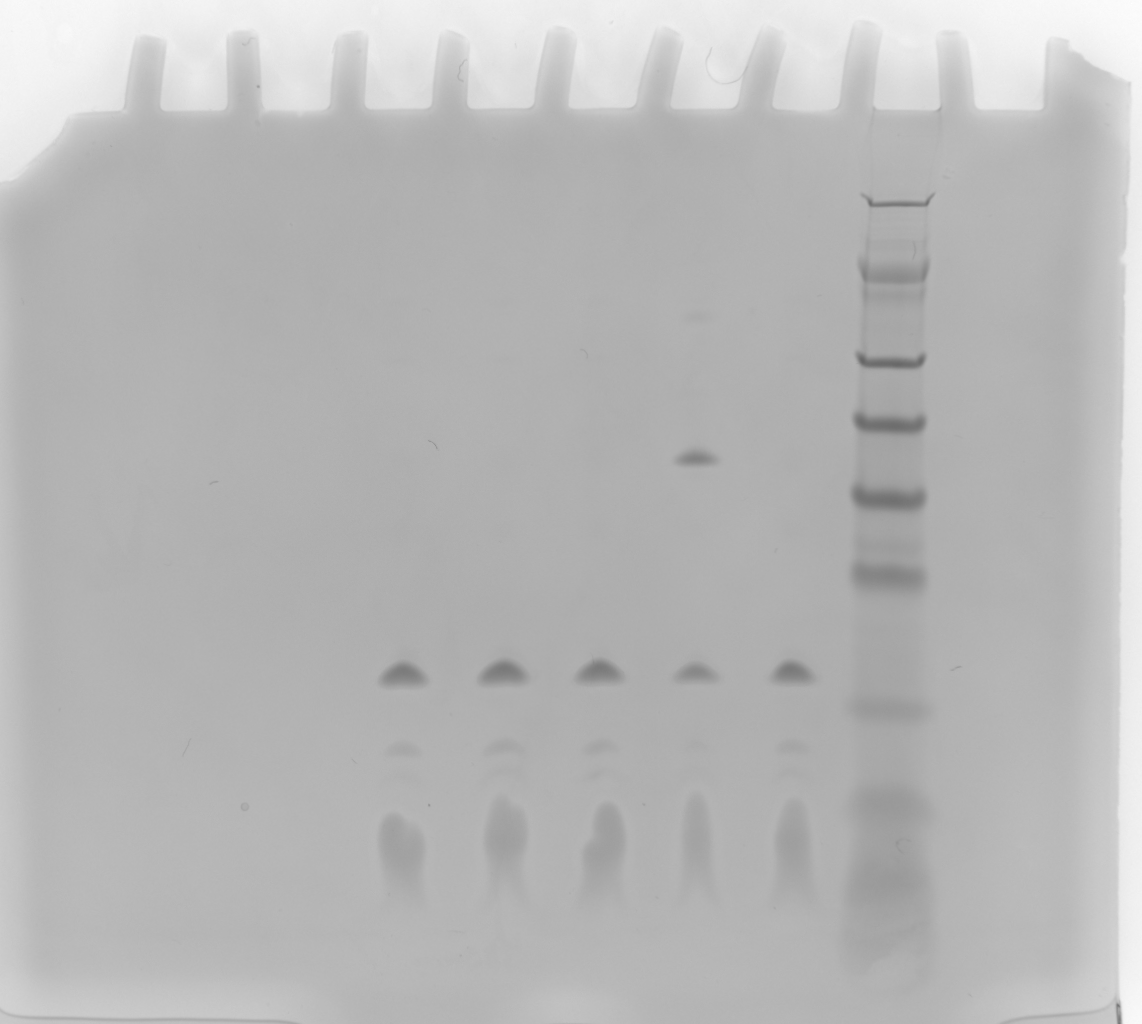

Supplement: Source data 1. [file elife-71938-data1.zip › Bera et al_Figure 1 Supplement 3_Source Gel 1.tif]

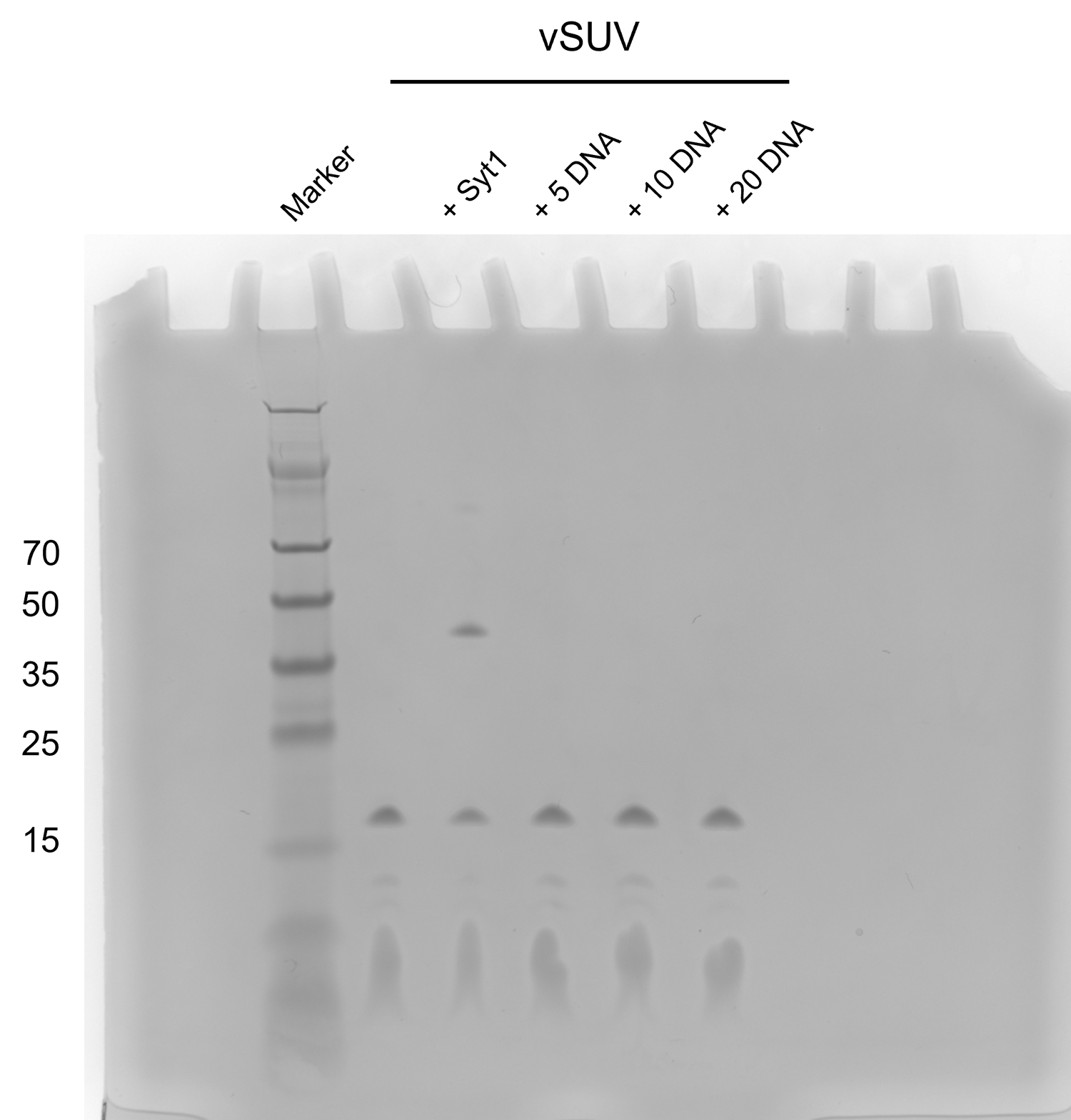

Supplement: Source data 1. [file elife-71938-data1.zip › Bera et al_Figure 1 Supplement 3_Source Gel 1_Labelled.tif]

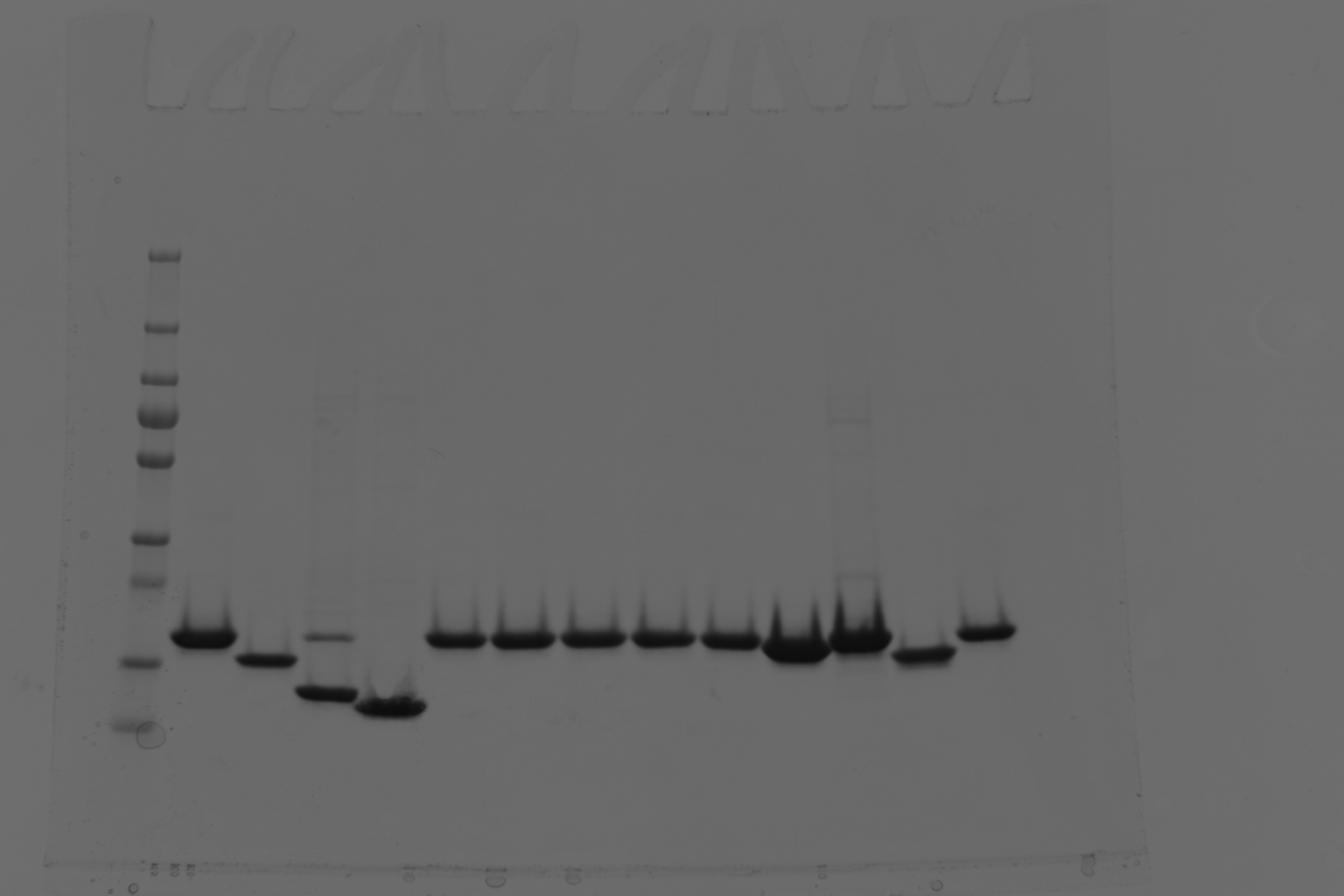

Supplement: Source data 1. [file elife-71938-data1.zip › Bera et al_Figure 1 Supplement 1_Source Gel 2.tif]

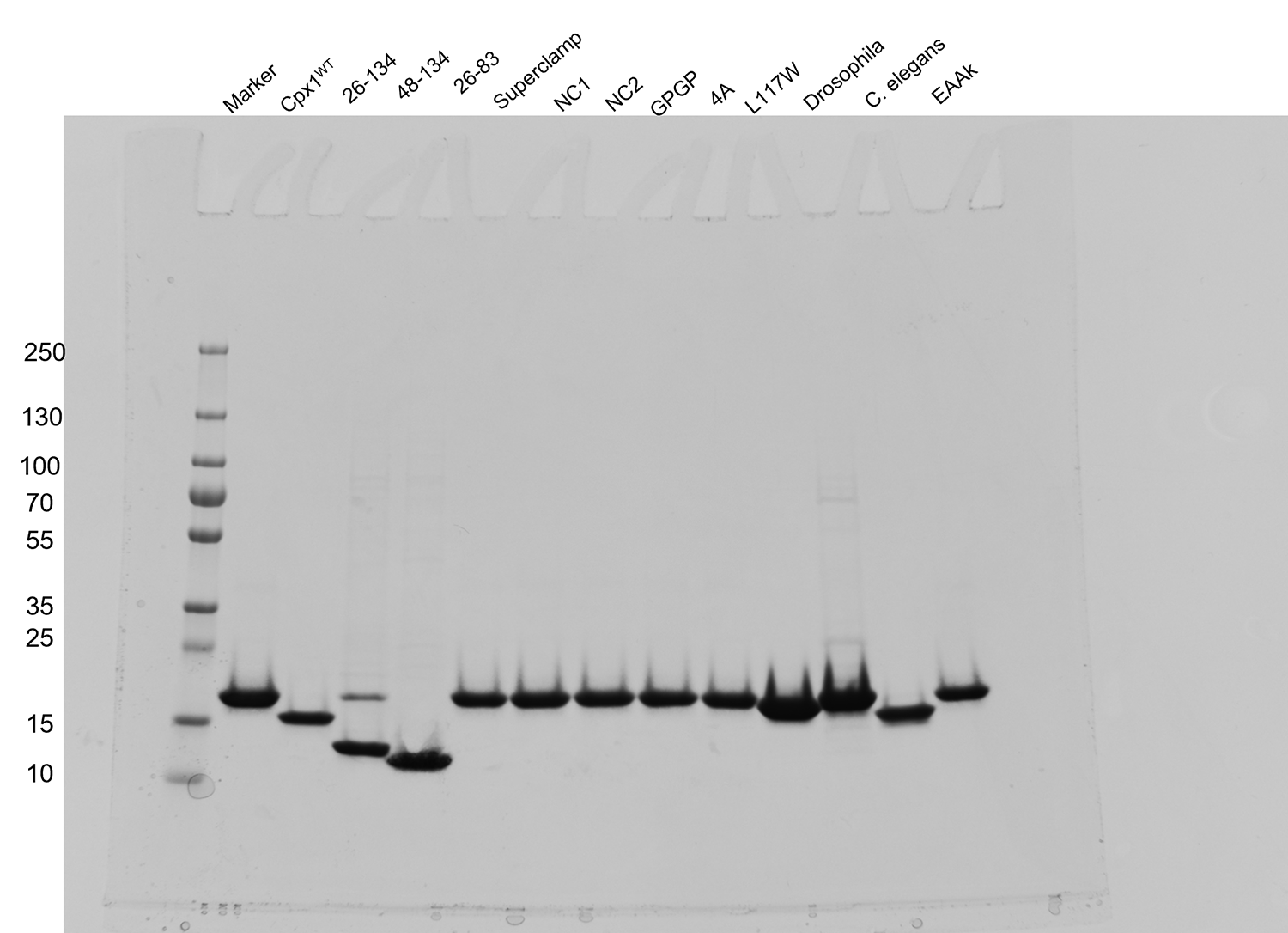

Supplement: Source data 1. [file elife-71938-data1.zip › Bera et al_Figure 1 Supplement 1_Source Gel 2-Labelled.tif]

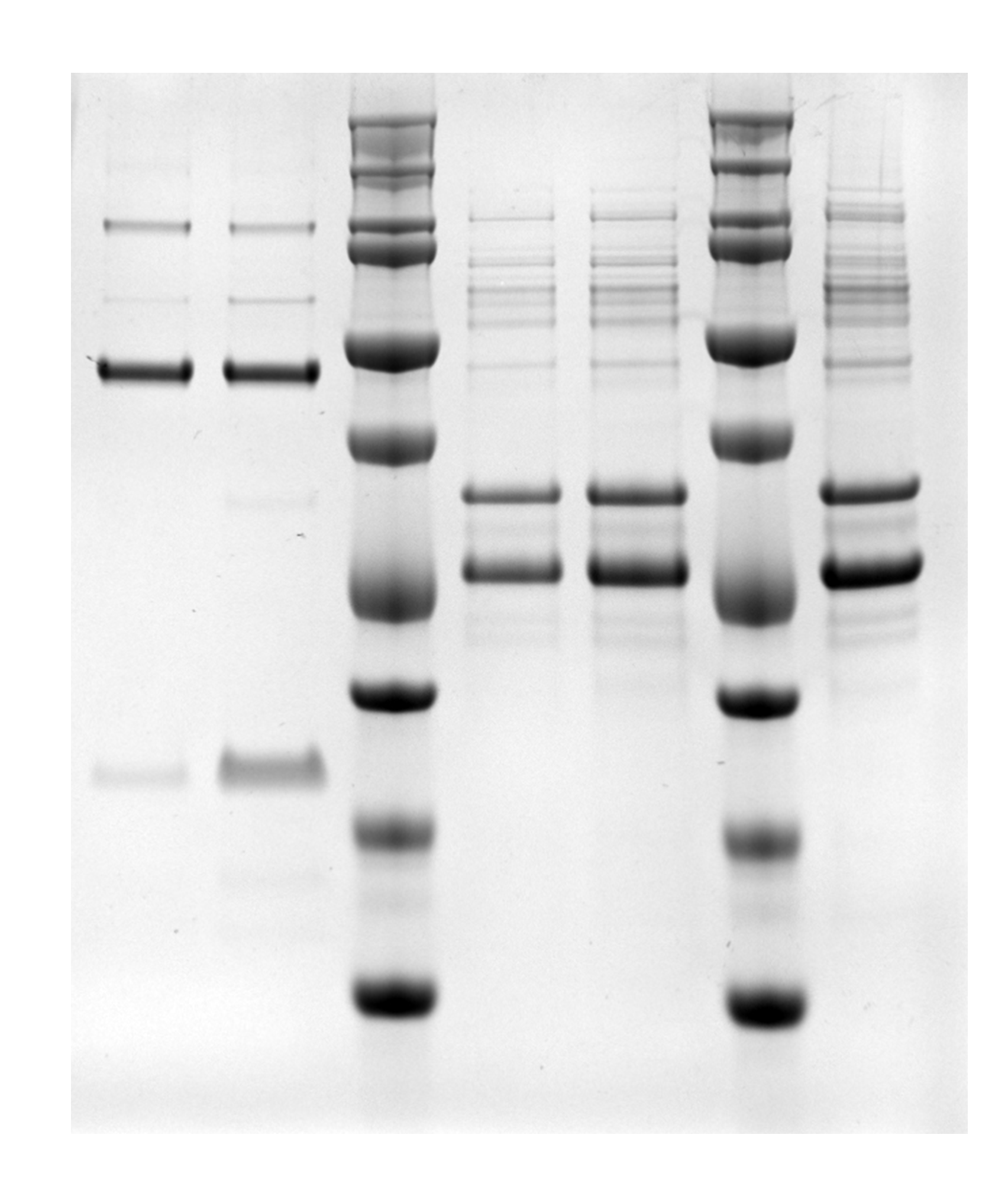

Supplement: Source data 1. [file elife-71938-data1.zip › Bera et al_Figure 1 Supplement 1_Source Gel 1.tif]

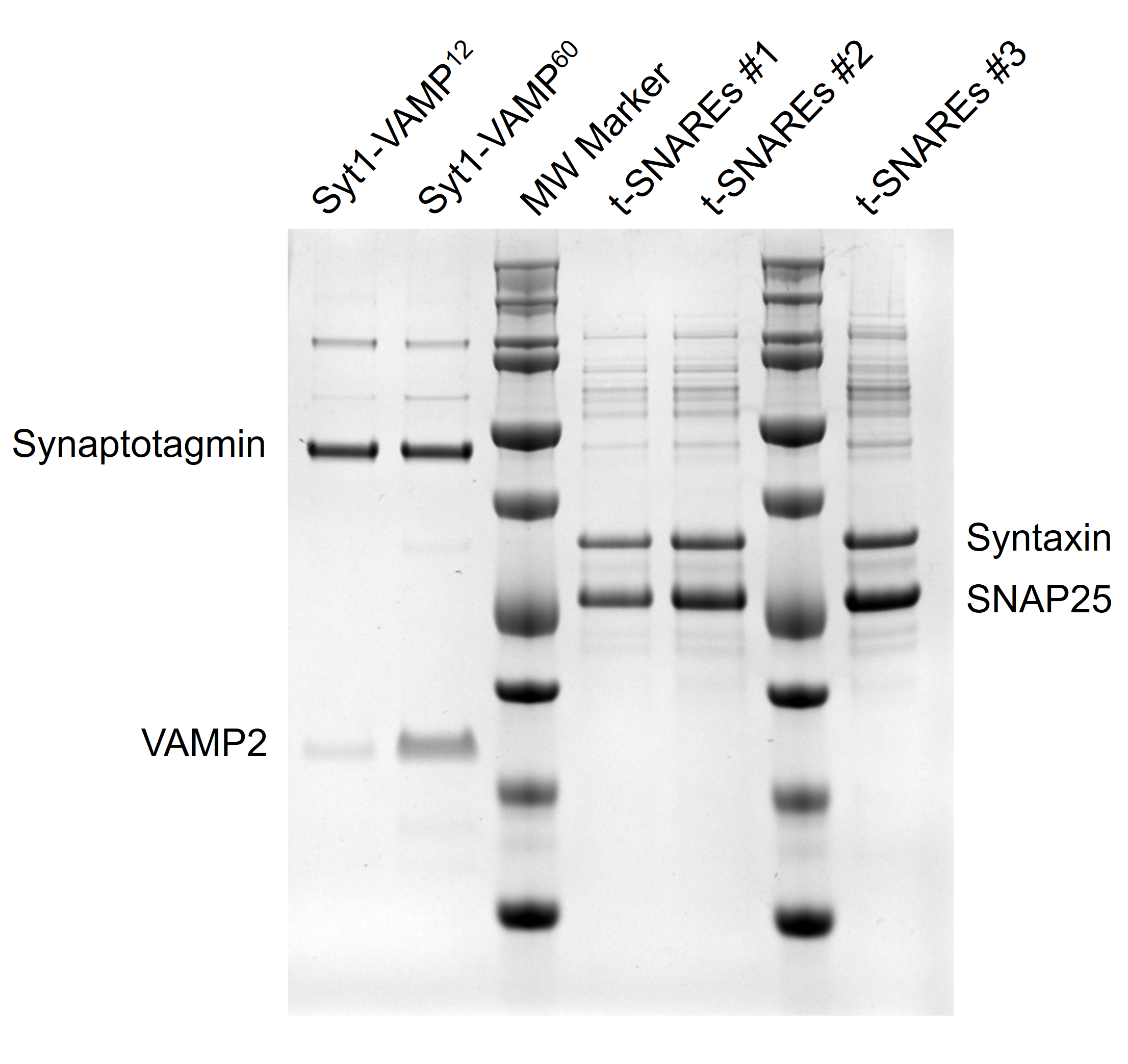

Supplement: Source data 1. [file elife-71938-data1.zip › Bera et al_Figure 1 Supplement 1_Source Gel 1-Labelled.tif]
